# Obstacles to returning to work with chronic pain: in-depth interviews with people who are off work due to chronic pain and employers

**DOI:** 10.1186/s12891-019-2877-5

**Published:** 2019-10-27

**Authors:** Mary Grant, Sophie Rees, Martin Underwood, Robert Froud

**Affiliations:** 1 Warwick Clinical Trials Unit, University of Warwick, Coventry, UK; 2 University Hospitals Coventry and Warwickshire, Coventry, UK; 3 Institute of Health Sciences, Kristiania University College, Oslo, Norway

**Keywords:** Chronic pain, Return to work, Qualitative interview

## Abstract

**Background:**

The global burden of chronic pain is growing with implications for both an ageing workforce and employers. Many obstacles are faced by people with chronic pain in finding employment and returning to work after a period of absence. Few studies have explored obstacles to return-to-work (RTW) from workers’ and employers’ perspectives. Here we explore views of both people in pain and employers about challenges to returning to work of people who are off work with chronic pain.

**Methods:**

We did individual semi-structured interviews with people who were off work (unemployed or off sick) with chronic pain recruited from National Health Service (NHS) pain services and employment services, and employers from small, medium, and large public or private sector organisations. We analysed data using the Framework method.

**Results:**

We interviewed 15 people off work with chronic pain and 10 employers. Obstacles to RTW for people with chronic pain spanned psychological, pain related, financial and economic, educational, and work-related domains. Employers were concerned about potential attitudinal obstacles, absence, ability of people with chronic pain to fulfil the job requirements, and the implications for workplace relationships. Views on disclosure of the pain condition were conflicting with more than half employers wanting early full disclosure and two-thirds of people with chronic pain declaring they would not disclose for fear of not getting a job or losing a job. Both employers and people with chronic pain thought that lack of confidence was an important obstacle. Changes to the job or work conditions (e.g. making reasonable adjustments, phased return, working from home or redeployment) were seen by both groups as facilitators. People with chronic pain wanted help in preparing to RTW, education for managers about pain and supportive working relationships.

**Conclusions:**

People with chronic pain and employers may think differently in terms of perceptions of obstacles to RTW. Views appeared disparate in relation to disclosure of pain and when this needs to occur. They appeared to have more in common regarding opinions about how to facilitate successful RTW. Increased understanding of both perspectives may be used to inform the development of improved RTW interventions.

## Background

The global burden of chronic pain is growing [1]. Chronic pain affects one-third to one-half of the UK population [2] and may have a major impact on ability to work [2, 3]. The commonest chronic pain is low back pain, which is the leading global cause of years lived with disability [4].

A cross sectional study of patients in Denmark with non-malignant chronic pain referred from general practice for treatment at a hospital multi-disciplinary pain clinic found evidence of reduced productivity and work capacity incurring costs to employers and society [5]. Back pain is one of the most costly conditions for which an economic analysis has been conducted in the UK and production loss is one of the key costs [6]. In 2016 it was estimated that ill health costs the UK economy £100 billion with musculoskeletal conditions being the second highest cause of sickness absence [7]. Unemployment was found to be significantly correlated with chronic pain in a US cross-sectional study and chronic pain was mainly attributed by respondents to low back pain and osteoarthritis [8].

In a meta-ethnography of 41 qualitative interview and focus group studies on the experience of RTW from the perspectives of people with chronic pain, and their employers, we found that managing pain, work relationships and making work place adjustments were described as being thought central to a successful RTW [9]. However, only five of the 41 studies were conducted with employers despite people with chronic pain emphasising the important influence of employer attitudes and knowledge on the RTW process [10].

Understanding the experiences and perspectives of both people with chronic pain and employers could lead to the development of better RTW interventions [7].

In this study our aim was to explore obstacles to, and facilitators of, sustainable RTW, as perceived by people with chronic pain and employers.

## Methods

### Research design, sample and recruitment

Working within an interpretative paradigm, a qualitative interview study was deemed the most appropriate and pragmatic way to explore participants’ perceptions and fulfil the research aim. People off work with chronic pain attending NHS pain clinics/pain management services or a Job Shop run by a Coventry city council were invited to participate in an individual semi-structured interview. The reason we chose these sites was to access a range of people with chronic pain from hospitals where they were seeking treatment for their pain and a non-medical centre (the Job Shop) where their primary focus was looking for work. We hoped this would provide a variety of different perspectives on obstacles to and facilitators of returning to work.

The Job Shop provides a wide range of support to city residents, of all ages, who are looking for work. Working with partners across the city it also houses an Employer Hub and actively works with employers to generate opportunities for local people [11].

In the NHS, potential participants were identified by clinicians (pain consultants and physiotherapists) when attending out-patient appointments or multidisciplinary pain management courses. In the Job Shop potential participants were identified by job coaches and through a database search. We included adults who were off work (unemployed or off sick) due to chronic non-malignant pain, and sufficiently fluent in English to consent to the study and be interviewed. Potential participants were given an information pack including an invitation letter, participant information leaflet, expression of interest form, declining form and a stamped addressed envelope by the clinician or employment coach. Potential participants returned the expression of interest form to the research team and a team member then contacted them by telephone to explain the purpose of the study and describe what the participant’s involvement would be. The researcher checked and confirmed eligibility, answered any questions the participant may have had about the study before arranging the interview where written consent was obtained.

Employers were recruited through an employer hub at the Job Shop, local Chamber of Commerce (lunch event) or through personal and professional contacts of the research team. Eligibility criteria were that they were in a senior position (for example Chief Executive Officer (CEO), managing director or senior managers in human resources or occupational health departments) of small, medium and large, public or private goods or service producing organisations and were sufficiently fluent in English to consent to the study and be interviewed. We targeted this range of establishments in order to explore views potentially influenced by the size and type of organisation. Employers were invited to participate face-to-face or by telephone or e-mail and were sent a participant information leaflet and consent form. The purpose of the study and the nature of their involvement were described to the employers by a member of the research team and they were given the opportunity to ask any questions they had about the study. Eligibility was checked and confirmed and interviews were arranged. Written consent was obtained at the interview.

The purpose of the interviews was to explore the perception of obstacles to, and facilitators of, RTW. All interviews were conducted by a study research fellow (MG) with postgraduate training and twenty years’ experience in qualitative research. All interviewees were offered a £20 shopping voucher as a thank you for their participation and because previous research shows this facilitates recruitment in studies of this nature [12].

We developed topic guides for the interviews (Appendix); for people with chronic pain and for employers, through a review of the literature [9] and discussion with patient and public representatives and the study team (including an employment support manager, General Practitioner (GP), health psychologist, and an occupational therapist). The patient and public representatives and study team were asked for their feedback on a draft version of the topic guides and some suggestions were made to improve them. These guides were used flexibly to allow for exploration of some topics in more depth depending on the individual being interviewed.

In addition to the development of the research aim, interview questions, and data collection methods were also based on previous research in this area and shaped by the mix of health professionals, non-health professionals and public and patient representatives in the study team. Moreover, part of the employer sample recruited was directly influenced by this team through their personal and professional contacts. However, none of the participants were personal contacts of the interviewer.

### Data analysis

We digitally recorded all the interviews and transcribed them verbatim. We then anonymised and analysed the data using the Framework method [13]. Transcripts were first read and re-read by reviewers (MG, SR and RF). After familiarisation, SR and MG independently coded a sample of transcripts (two employers and two participants with chronic pain), and during this process inductively developed a provisional framework that was relevant to the a priori study objectives. A qualitative software package, NVivo 11 (QSR International, Victoria) was used to assist with data management and analysis. This software allows key parts of transcripts to be tagged and grouped into themes and enables the formation of framework matrices. MG, SR and RF then met to discuss the suitability of an analytical framework and to refine the provisional framework, which was then applied through charting the remaining data into the proposed framework matrix [14]. Any difficulties with charting were noted and discussed between the researchers, with modifications being made to the framework as appropriate. Examples of modifications included merging and renaming themes where researchers had used different words for the same concept. After charting the data the researchers discussed interpretations of the data and explanatory models were constructed, where useful, to explain any associations between identified characteristics and the views of people in pain and/or employers .

Ethics approval was granted by the London-Stanmore Research Ethics Committee (Reference 17/LO/0919).

## Results

Interviews took place between August 2017 and February 2018 and were conducted in participant’s homes or employers’ work places with four exceptions. One participant with chronic pain chose to be interviewed in a public library and a room was booked for this interview to ensure confidentiality. Two participants with chronic pain chose to be interviewed at the Job Shop where they were clients. One employer chose to be interviewed at the university where the research team were based.

Research staff distributed 240 information packs to staff at the recruitment sites for people with chronic pain and 149 were given out to potential participants. Of these, 54 expression of interest forms were returned to the research team and 19 of these were from people who were interested in finding out more whereas 35 were from those who were not interested. Four of those who expressed an interest decided they did not wish to participate after finding out more. Fifteen people with chronic, non-malignant pain were interviewed. Six were recruited from the Job Shop and the rest via NHS pain services. Eight females and seven males with chronic pain in a variety of locations including back, neck, arm, hip, knee and ankle were interviewed. Pain was mainly caused by injury and osteoarthritis. Characteristics of participants with chronic pain are summarised in Table 1.

**Table 1:** Characteristics of participants with chronic pain

Study number	Age range	Gender	Type/Cause/ Location of pain	Duration of pain	Work situation	Site recruited from
PI01	35–40	Male	Injury to neck while using rowing machine in gym leading to painful left shoulder and arm with little function (nerve damage) Back pain	2 years	Unemployed since injury due to pain as unable to do previous job (warehouse work)	NHS Pain clinic via physiotherapy
PI02	65–70	Male	Back injury at work – threw a pallet in anger about a colleague being bullied and his back went into spasm and now has pain from back down to toes and pins and needles – diagnosed with sciatic nerve compression	Constant pain since 1992 (25 years) but had back problems before that	Retired now – retired earlier than he would have liked due to back pain	NHS Pain clinic via physiotherapy
PI03	30–35	Male	Accident with machinery at work – caused back pain linked with hernia of spine at L5 and degeneration of discs	4 years	Suspended from work due to number of episodes of sickness absence because of pain	NHS Pain clinic via physiotherapy
PI04	60–65	Female	Hip replacement in 2004 then in 2007 started getting spasms in left thigh from the groin down to the knee but nothing found on scans and X –rays Back pain due to osteoarthritis - diagnosed with stenosis of spine & five bulging discs from base of spine to waist	10 years	Off sick from work due to pain – waiting for meeting at work to discuss return	NHS Pain clinic via physiotherapy and attended pain management programme
PI05	30–35	Female	Lower back pain since age of 13 linked with childhood abuse – diagnosed with degenerative arthritis of coccyx, two bulging discs Torn disc of spine two years ago – following severe coughing caused by respiratory infection	18 years	Became self-employed due to back pain causing difficulties and sickness absence in previous job (residential care work)	NHS Pain clinic via physiotherapy and attended pain management programme
PI06	35–40	Female	Fell over and twisted ankle – fracture not diagnosed initially but diagnosed sometime after injury when already healed Complex Regional Pain Syndrome diagnosed later	5 years	Unable to continue with jobs due to pain so now unemployed and claiming benefits	NHS Pain clinic via consultant physician
PI07	60–65	Male	Pain in feet at night due to nerve damage secondary to diabetes Pain in leg linked with fall – found two small metal pieces in leg (maybe as a result of previous motorbike accidents) Pain in left arm coming from neck into shoulder	Since heart attack 2 years ago	Unemployed. Went onto Employment and Support Allowance (ESA) after heart attack as no longer able to do previous job (fork lift truck driver) and was recently moved from ESA to Job Seekers Allowance (JSA) following health assessment	Job Shop
PI08	55–60	Male	Congenital disability that affected one side of body and wore a calliper as a child. Has had a number of operations on his affected foot to straighten it as used to walk on tiptoe. Has pain in both sides of his body and especially the foot that was operated on	Since childhood	Unemployed – recently moved from ESA to JSA following health assessment	Job Shop
PI09	20–25	Female	Pain (burning and stabbing pain) from knees downwards – worse at night and in the morning – cause unknown – nerve tests inconclusive Morton’s Neuroma in feet cause pain Pain linked with Chronic Fatigue Syndrome (CFS)	Pain in legs - last year and a half Pain in feet −1 year Pain linked with CFS – 6 years.	Unemployed – on ESA but does not get any money as partner works more than 20 h	NHS Pain Clinic via clinical psychologist in Multi-Disciplinary Team (MDT)
PI10	50–55	Male	Fractured spine – internal fixation and has five degenerating vertebrae	13 years	Unemployed – on benefits	NHS Pain Clinic via consultant physician
PI11	50–55	Female	Lower back pain linked with previous job	Many years of intermittent back pain but more severe in last 6 months	Unemployed – unable to do previous job due to pain	Job Shop
PI12	45–50	Female	Arm pain linked with tennis elbow	8 years	Unemployed due to pain	Job Shop
PI13	50–55	Male	Slipped disc at bottom of lumbar spine – work related injury Trapped nerve in left shoulder	Back pain – 7 years Shoulder pain – 10 years	Unemployed due to difficulties in previous job linked with pain and disability	NHS Pain clinic via physiotherapist
PI14	25–30	Female	Knee pain – ligament damage exacerbated due to fall	Couple of years ago	Unemployed	Job Shop
PI15	55–60	Female	Osteoarthritis causing knee pain One partial knee replacement	A few years Partial knee replacement one year ago	Unemployed – made redundant five months previously when store where she worked closed down	Job Shop

The Employer Hub sent out 300 emails with information leaflets attached, 50 information packs were given out at a Chamber of Commerce lunch event by research staff, six personal contacts of the research team were approached and fifteen professional contacts of the team were invited to participate via telephone and email. Ten employers were interviewed. Six were recruited from small, three from large organisations and one from a medium sized organisation, six private and four public and a mix of seven service and three goods producing. Employer roles included CEOs, managing directors, human resource managers and an occupational health lead. Four were personal contacts and five were professional contacts of the research team and one was recruited via the Chamber of Commerce lunch event. Employer characteristics are summarised in Table 2.

**Table 2:** Characteristics of employers

Study number	Interviewee role	Employing sector	Size of company/ organisation	Number of employees	Nature of business
EI01	Chief Executive Officer (CEO)	Private/ Service producing	Small	10 employees	Construction industry
EI02	Head of Inclusion, Engagement and Wellbeing (nurse by background)	Public/ Service producing	Large	10,000 employees	Acute NHS Trust
EI03	Practice manager	Public/ Service producing	Small	17 employees	NHS GP
EI04	Occupational Health Lead (GP by background)	Private/ Goods producing	Large	100,000 employees globally 13,500 permanent UK staff 5–6000 UK complementary workers	Health care company – research and manufacturing
EI05	Company director	Private/Service producing	Small	12 employees	Digital marketing agency
EI06	Senior Human Resources (HR) Manager for employee relations and wellbeing	Private/Goods producing	Large	35,000 employees	Car manufacturing company
EI07	Owner/manager	Private/Service producing	Small	9 employees	Gym and personal training studio
EI08	Managing director and key account manager for automotive business	Private/Goods producing	Large/ Small	14,000 employees globally 7 UK employees	Tier 1 supplier to the automotive industry (97%) and industrial business (3%) UK – Sales and distribution
EI09	HR and Facilities Manager	Private/Service producing	Medium	120 employees	Private equity partnership offering legal services/ advice
EI10	Manager (and past experience as HR manager in large private companies)	Public/Service producing	Small	8 employees	NHS GP practice

Recruitment and interviews took place over an eight month period. The duration of the interviews for people living with chronic pain ranged from 17 to 81 min and the mean was 40 min. For employers the range was 15 to 69 min and the mean was 37 min.

Framework themes are listed in Table 3 and a selection of these themes, described and supported by quotations, will be presented below. Table 3 provides an overview of the views of people with chronic pain and employers separated into the first two columns. The third column in the table comprises shared perceptions. In the following section we summarise findings from employers and people with chronic pain together noting both similarities and differences.

**Table 3:** Framework

Theme	People with chronic pain	Employers	Common themes
Obstacles to return to work	Psychological obstacles - Fear and anxiety - Fear of pain becoming worse - Worried about what others think (of me) - Negative experiences in the past Pain related obstacles - Unpredictability of pain - Reduced mobility - Sleep difficulties Financial and economic obstacles - No financial incentive - Competitive job market - Cost to company (productivity) Educational obstacles - Lack of qualifications - Challenges of dealing with technology Work history obstacles - Being out of work for a long time - Gaps in CV Work environment and nature of work - Pressure of work - Finding suitable employment - Not being able to pull your weight - Feels work offered is unsuitable - Unable to fulfil job requirements - Difficulties fulfilling previous role Employer related obstacles - Inflexible manager - Perceived discrimination and unfair treatment - Employer unwilling to make adjustments - Being judged by potential employers - Unsupportive manager	Attitudinal obstacles - Attitude of person with chronic pain - Attitudes of others towards people with chronic pain - Stigma Managerial obstacles - Managers lack of people skills - Managers lack of understanding about pain Organisational context - Reasonable adjustments not possible or affordable - Dismissal if unable to redeploy - Ageing work population and increased demand for redeployment - Conflict between occupational health and employer about what adjustments are reasonable and realistic - Austerity and limited rehabilitation & resources in NHS - Culture and size of organisation - Fairness and potential for conflict with colleagues - Fear of litigation Capability related obstacles - Ability to do the job - Poor performance - Physical demands of job - Absence - Hospital appointments	- Lack of confidence - Reliability - Travelling to work
Facilitators of return to work	Workplace adjustments - Temporary job swap - Finding suitable job - Flexibility from employer - Small family run business Good working relationships - Good relationship with employer, manager and colleagues - Understanding manager and colleagues - Being listened to - Being treated as a person and not a disability - Help from colleagues Education - Education for managers about pain - Understanding of capabilities Preparing for RTW - Interview preparation support - Placements - Support looking for a job - Training - Trial period Interventions - Effective pain relief - Specialised physiotherapy	Workplace adjustments - Accommodation of hospital appointments - Reducing physical demands of job - Support, training and investment from employer Interventions - Provision of ergonomic work space - Coaching - Individually tailored to support to return to work - Private medical insurance provided by company - Occupational health provision Cultural factors - Caring and compassionate company - Changing culture and attitudes to employment for people with disabilities and chronic pain - Political will and coordination of Department of Work and Pensions and Department of Health	Workplace adjustments - Making changes to job and hours - Adaptation of the environment or provision of equipment - Making reasonable adjustments - Phased return - Redeployment - Working from home - Reduced hours - Taking breaks - Light duties - Help with travelling to work (Access to work or providing transport) Interventions - Access to cognitive behavioural therapy and counselling - Access to physiotherapy
Disclosure of chronic pain to employer	- Not disclosing during application or interview process - Fear of not getting an interview or job - Fear of consequences of not disclosing - Fear of losing job	- Disclosure so employer can make reasonable adjustments - Disclosure to access interventions in the workplace - Disclosing to occupational health not recruiting manager - Understanding and accommodating non-disclosure	- Need to be open and honest

### Perceived obstacles to return to work

People in pain perceived an array of obstacles including psychological, pain related, financial and economic, educational, work history and those related to the work environment, type of work and employer. Employers, on the other hand, were concerned with attitudinal issues, culture and size of the organisation, managers lacking understanding and people skills, job demands, performance and scenarios when reasonable adjustments are not possible or affordable.

#### Psychological obstacles

Psychological obstacles described by people with chronic pain were mainly fear and anxiety. Being out of work for a long time and being in pain had made the notion of beginning to work again overwhelming and frightening.

*‘Maybe just that because obviously I’ve been in pain for quite a while and I’ve been a bit out of, like, normal society that just, I think things are more anxious than obviously before it wouldn’t have been a problem at all. It’s all new things and stress. Yeah, it’s just massive. I wouldn’t know where to start on my own.’ (PI09 – 6 years of pain - unemployed)*

‘F*ear stops you doing things and fear of putting people's backs up, cause it does happen.’ (PI15 – a ‘few years’ of pain - unemployed)*

Both people with chronic pain and employers perceived lack of confidence as a key psychological obstacle to work return. They explained that it is a highly influential obstacle that is linked with low self-esteem and negative thinking which can prevent someone being able to interact with the world around them because they have not been in the workplace for some time.

*‘I think a lot of it may be confidence in themselves whether they deem they can still do tasks in hand, interaction possibly with people, just in general and to talk with people.’ (EI01 – Employer – CEO small private company)*

*‘It brings about quite a lot of negative imbalance, in thoughts and about yourself, your esteem goes, your confidence goes down, you withdrawing from society and it only gets worse.’ (PI06 – 5 years of pain - unemployed)*

#### Attitudinal obstacles

Employers had varying views about how the perceived attitudes of people with chronic pain might present obstacles to returning to work. Some employers drew on cultural narratives about people with chronic pain as ‘lazy’, whereas others suggested some people feel unhappy being unable to contribute at the same level as they previously could.

*‘Well, some people, to be honest, it’s just laziness. Like, in the way ‘cause they’ve had that time off and they’ve been paid to have that time off they don’t, you know, they can’t be bothered, they can’t, some people, this is being honest.’ (EI07 – Employer – small private company)*

*‘And it’s an issue of personal pride to them, they feel they can’t keep up with what their colleagues would expect from them, or their employer would expect from them.’ (EI10 - Employer – manager in small public sector organisation and experience of HR role in large private sector organisation)*

Authenticity with respect to pain conditions was questioned by two employers. One said that people with pain were just lazy, as quoted above, and another questioned whether a pain condition was the real reason why an employee left the organisation. Having had surgery was seen as legitimising chronic pain, whereas something invisible such as chronic back pain garnered less sympathy.

*‘If you’ve had surgery then, yeah, you get, you get more sympathy because, well, people don't give you a new knee if you haven’t actually got something wrong, do they? But you can be taking painkillers for a bad back and it ... how bad is a bad back? You know, I think some illnesses are classed as lazy people’s illnesses.’ (PI15 - a ‘few years’ of pain - unemployed)*

The above participant also pointed out scepticism and lack of sympathy towards people with fibromyalgia which she grouped with chronic fatigue syndrome due to these conditions not being visible.

#### Capability and reliability

Employers and people with chronic pain described their doubts in relation to capability and reliability. A primary concern of employers was whether chronic pain would prevent a person being able to fulfil the demands of their job.

*‘Our job out on site is a lot of physical aspect of it … Whether they’d be able to actually physically undertake the job would be an issue as well as obviously if it was more office based it’s you know what state of mind they are in ‘cause we have to do quite a lot of interaction with members of the public.’ (EI01- Employer – CEO – Small private company)*

This was also reflected in the interviews with people with pain, who were worried they would not be able to fulfil the job requirements or that they would try to do so and this would cause the pain to worsen.

Both employers and people with chronic pain expressed concerns about reliability.

*‘I’ve had individuals who’ve worked for us before and their time keeping and having days off and being unreliable and any business would say that … unreliability whether it’s … no matter what trade, it’s reliability is the key thing.’ (EI01 – Employer - CEO small private company)*

*‘And it’d be like, you wouldn’t get 24 hours’ notice that I’m not coming in. It’ll be in the morning, “I can’t make it today,” you know. So it’s that a reliability issue as well. That’s a big thing.’ (PI10 – 13 years pain - unemployed)*

Employers were concerned with potential increased workload on other employees and managing potential conflict amongst their employees as a result.

*‘A phased return to work, that does put quite a strain on the other people in the teams and that can sometimes develop into animosity. So from my concern is not just dealing with the individual and trying to get them back into the workplace, it's also the impact on the other people that are left here to do the work as well.’ (EI09 - Employer – HR Manager - Medium sized private company)*

In a similar vein, people with chronic pain were also concerned that they may be a burden on their colleagues and worried what people would think of them.

*‘I feel if I can’t pull my full weight … that’s going to be a difficulty for me to overcome, if I can’t do the job I am paid for I’m gonna find that a bit difficult … but I know that I’ve got to be careful!’ (PI04 – 10 years pain – off sick)*

*‘And there were times when we were overrun and we had ... “I’m sorry, I can’t physically do it.” And that makes you feel that you’re letting the team down.’ (PI15– a ‘few years’ of pain - unemployed)*

It was evident that people with chronic pain have a sense of pride in doing the job to the best of their ability and felt their pain prevented them from achieving this.

A number of people, no longer able to do the physical work they did previously, faced the additional obstacles of not having the administrative or computer skills or qualifications to change to a different kind of job.

*‘Because I can’t use the computer or anything like that, them sort of jobs are out of the question ... you know. It’s no good to me.’ (PP10– 13 years of pain - unemployed)*

#### Organisational context

Organisational context was thought to influence obstacles to RTW. For example, one company provided training to ensure managers felt comfortable discussing with employees the adjustments they required *(EI04 – Employer - Occupational Health Lead – Global health care company)*. Two large public and private sector organisations were able to offer in-house rehabilitation to support RTW, but smaller organisations were restricted by their more limited resources. A human resources manager from a medium sized private company who also had work experience in the public sector highlighted that smaller companies tend to be more risk averse in terms of employing people with health conditions.

Even within organisations, there may be variation in the willingness of different managers in the same organisation to make reasonable adjustments.

*‘I think the key thing is the organisation understanding what ‘reasonable adjustment’ means because I know of some cases where we’ve gone totally above and beyond and the person hasn’t accepted that they can’t do the work and we need to help them realise that they can’t do the work so from an organisation perspective. I often say to people it doesn’t say ‘bank of [organisation name] outside’ we do not have untold resources and money to make adjustment after adjustment after adjustment for somebody who really can’t do the work that they were employed to do but then we’ve also got managers who don’t even want to make any adjustment!’ (EI02 – Employer - Head of Inclusion, Engagement and Diversity, Large Public Sector Organisation)*

Some employers expressed frustration with the way outsourced occupational health services sometimes made unrealistic recommendations for adjustments as illustrated by the quotation below

*‘Most people in occupational health are there because they genuinely believe in what they’re doing. But I think they are very focused on the individual in front of them and their duty of care to that person and I can only praise that. I don’t necessarily think that translates into a great degree of reality. Occupational health specialists employed by companies have a much healthier dose of realism, because they know who’s paying their wages’ (EI10 - Employer – manager in small public sector organisation and experience of HR role in large private sector organisation)*

UK welfare reforms, the introduction of more ruthless absence management policies, and an ageing workforce were some aspects of the socio-political context which added pressure to the limited resources of organisations, limiting their ability to support people with chronic pain.

Concern was also expressed about fulfilling their legal responsibilities as an employer as illustrated below.

*‘I think some of the things that might make people feel a bit anxious … there's lots of focus around what employers need to do when there's disabilities or conditions that need to be managed.’ (EI06 – Employer - Senior Human Resources Manager – Large manufacturing company)*

### Facilitators of return to work

#### Preparing for return to work

People with chronic pain who were unemployed described belief that support to prepare for interviews and look for a job would facilitate RTW.

*‘ … but if I was to apply for a job and go for an interview, I don’t even know how they’d do it anymore, so there’s a fear of that, you don’t know if you are prepared, maybe that’s something that could be implemented in helping, like doing fake interviews to show them the process all over again … get used to it.’ (PI03 – 4 years pain – suspended from work due to sickness absence)*

One participant who was off sick suggested a trial period would be very helpful in addressing the uncertainty around how work would affect their pain condition.

*‘I think to give them the chance to do it … the employer to actually say “if you feel you want to come back now we’ll give you a trial period over three months or whatever and we’ll take it from there” I think that would help me because I’m thinking “will I ever go back to work?” and then it’s “do I want to go back to work”?’ (PI04 – 10 years pain – off sick)*

#### Workplace adjustments and interventions

There was considerable commonality in the perceptions of people with chronic pain and employers about what would facilitate a successful RTW, especially in terms of workplace adjustments. Examples given included altered or reduced hours, taking breaks, phased return, light duties, working from home, help with travelling to work, and redeployment if needed.

Participants described the benefits of having understanding and flexible colleagues or managers who made adjustments unofficially to help the person with pain.

*‘There is light duties you can do, they just tell you you’re not allowed but luckily enough with him he understood the score he know how much pain I was in so he’d be like “right I’ll do your job you just go and jump on the cardboard”.’ (PI03 – 4 years pain – suspended from work due to sickness absence)*

People with chronic pain emphasised the importance of a good working relationship with managers and colleagues and appreciated them listening and being understanding about pain. Some participants suggested that education for managers about pain and a greater understanding of the employee’s capabilities would be helpful.

*‘I think the people in charge, the management, they should take a course on people with back problems, so they know what they’re going through. Because they don’t ... do you understand what I mean? If I’d had a manager that knew the problems I’ve got .. I’d have felt more at ease and more comfortable working there.’ (PI13 – 10 years of pain – unemployed)*

The accommodation of taking time off work to attend hospital appointments was contentious for some small employers but supported by others.

Some employers suggested processes such as providing an ergonomic work space, occupational health, private medical insurance, and coaching.

*‘We also offer access to coaching for some individuals because we found that coaching can be really beneficial for some people and so the idea is very much that the nurse, as the case manager, pulls in and pulls on these resources and guides and signposts and we do our best to try and enable people to come back to work.’ (EI04 – Employer - Occupational Health Lead – Global health care company)*

Coaching was seen as a more attractive option than counselling because it is more akin to mentoring and can encompass career development while simultaneously supporting people returning after a period of absence, and thus it does not carry the same stigma as counselling. In this particular company it has been called ‘Returners Coaching’ and covers people coming back from illness, surgery, maternity leave or after absence for other reasons.

Workplace access to interventions such as cognitive behavioural therapy, counselling and physiotherapy were also cited by both groups as enablers, although one participant stressed that for her the physiotherapist needed to be specialised to be effective.

### Disclosure of chronic pain to employer

We asked all employers and people with chronic pain about disclosure of pain. Just over half the employers interviewed felt strongly that people should be open and honest and disclose their chronic pain. They were mainly from small public and private sector organisations. Some employers felt that non-disclosure would result in a sense of being deceived and would jeopardise trust.

*‘Personally if somebody didn’t tell me then I employ them and three months later they are off work for six to eight weeks with an existing condition I just think it’s not fair’ (EI03 – Employer – small public sector organisation)*

Another employer suggested that failure to disclose could lead to dismissal if the condition impacted on the person’s ability to fulfil job requirements.

*‘If they’ve only been there recently you make a decision to say “hold on there a second you should’ve told me this … it’s not working out I think we need to shake hands and move on!”’ (EI05 – Employer – Small private company)*

However, employers did generally understand employee’s lack of disclosure as shown in the quotation below.

*‘perhaps pressures from work, you know, ‘cause someone may have an illness and might be reluctant to kind of talk about it or feel as though that they can’t take time off for various reasons..so that’s sort of something you need to be mindful of’. (EI08 – Employer – Large private company)*

Other employers said disclosure was important so they could make reasonable adjustments for employees or enable access to workplace interventions. In larger organisations, employers explained that people can disclose their health conditions in confidence to occupational health departments, keeping this process separate from the recruiting manager and thereby protecting an employee from discrimination.

Two thirds of the participants with chronic pain reported that they have not or would not disclose their pain to an employer. Reasons for this included fear of not getting an interview or job, and fear of losing a job.

*‘You feel like you wanna approach them and tell them but you also feel like you will be ridiculed for it so they’ll look at you as a hindrance and think “right then we’ve gotta look at somebody else now because he might not be able to do this job for much longer” and then it can get to a point where, as I say, you have time off and they think “right search for someone get them trained and we’ll get rid of him” that’s how you feel … you feel like you are always on eggshells so you can’t do much, you are always worried!’ (PI03 – 4 years pain – suspended from work due to sickness absence)*

Motivators for disclosing included a moral obligation to disclose, and fear of losing their job as a result of not disclosing.

*‘I wanted to be straight with him. I didn’t want no lies.’ (PI07 – 2 years pain - unemployed)*

*‘Well, I, I was going by the, the idea of, if you don’t tell them, they can sack you. So I always go to the interview and say, “Oh, by the way, I suffer with this, this and this.” Because that way, they, then know that you’ve got a problem.’ (PI13 – 10 years pain - unemployed)*

## Discussion

### Main findings

Our interviews suggest that some perceptions of obstacles to RTW may be shared by employers and people in chronic pain. For example, lack of confidence due to a period of absence from work, and concerns about reliability were both raised by the two groups. However, there were some indications of divergence in other issues. For example, employers may more uniquely perceive obstacles to span domains of attitude of employees and conflict with occupational health about what constitutes a reasonable adjustment. In contrast people in chronic pain may more uniquely perceive obstacles to span domains of psychological obstacles, for example, fear and anxiety, sometimes related to negative experiences in the past, and may be particularly concerned about what co-workers and managers think about them. Inflexible, unsympathetic managers were seen as an obstacle. Employers were concerned about managing increased conflict between their other staff due to perceptions of injustice and increased workload. More than half the employers felt strongly that full, early disclosure was important and equated this with an employee being open, honest and trustworthy. But two-thirds of people with chronic pain said they would not disclose for fear of not getting a job or losing a job. However, employers were generally able to understand why people might not disclose, and a number of participants felt a moral obligation to disclose.

There was greater commonality of views between employers and people with chronic pain about how to facilitate a successful RTW including changes to the job or work conditions (e.g. making reasonable adjustments, phased return, working from home or redeployment). People with chronic pain also wanted help in preparing to RTW, education for managers about pain and supportive working relationships.

### Implications

There is some common ground between potential employers of people with chronic pain and those who are unemployed due to chronic pain which could be built upon when implementing interventions to enable RTW. For example, facilitating improvements in manager understanding as part of the intervention and support for people in chronic pain to help them to manage fears, negotiate workplace adjustments, discuss and disclose chronic pain, and open discussion surrounding the likelihood of fulfilment of job requirements may help sustained RTW. The introduction of a case manager to mediate between the employer and employee and provide support to both parties may assist with ensuring a good working relationship and enhance RTW success.

### Comparisons to existing literature

Many of the obstacles our participants with chronic pain identified are reported elsewhere in the literature [9, 15–19] e.g. lack of collaboration and understanding from employers [16, 19], lack of support, [16, 17] and pain-related issues [15, 18]. However there are few reports of employers’ perceptions. Like the current study, previous employer studies were conducted in a mix of small and large public and private sector organisations [20–24]. The context of economic crisis, restructuring, workforce reduction and subsequent impact on ability to make workplace accommodations has also been highlighted elsewhere [20, 21]. The growing challenge for employers in managing the chronic, recurring or fluctuating symptoms of an increasingly ageing workforce is recognised [25].

A lack of employer understanding of chronic pain could impact negatively on someone’s ability to RTW. It may result in unwillingness to make reasonable adjustments or even to employ someone with chronic pain in the first place. Not being believed and being judged were two themes shown to influence work relationships and RTW in our recent meta-ethnography [9] and this battle for legitimacy in the work context has been described in a previous qualitative review [3]. The societal narrative of disabled people as lazy, driven and reinforced by the media in the context of government welfare reform, appears to influence employer attitudes [26]. While this is a general for people with disability, and not specific for people with chronic pain, there is some suggestion that societal views are general. The UK legal definition of disability [27] is that a person who has a physical or mental impairment that has a ‘substantial’ and ‘long-term’ negative effect on your ability to do normal daily activities. Furthermore, the Department of Work and Pensions [7] discuss strategies to helping people with chronic pain in the context of helping disabled people. Paradoxically, there is also a culture of fear amongst some employers about not fulfilling their legal responsibilities to employees with disabilities highlighted in the current and previous research [28]. This could be a contributory factor in managers feeling uncomfortable discussing health issues and reasonable adjustments with their staff as reported in the current study. Stigma may impact on the confidence of individuals with chronic pain to negotiate working conditions that are sustainable. Fear and anxiety was sometimes attributed by participants in this study to past negative experiences including being bullied at work and potential job loss. Loss of confidence in relation to RTW, has been reported in two previous qualitative interview studies of people struggling to manage chronic pain [15, 29].

Other work has identified the importance of work adjustments as factors ‘pulling’ people back into work [30]. In our study, participants described how being in pain left them feeling isolated from wider society, including the working world, and this made them feel less confident when they considered returning to work.

Employers in previous studies complained that they were provided with insufficient detail and clarity about activity restrictions by occupational health staff and that unrealistic recommendations were difficult to implement, [21, 23] echoed in our findings. On the other hand, other research suggests that occupational health can be seen by employees with low back pain as being on the side of the organisation rather than the employee [31]. An occupational health physician in our current study described the use of coaching to assist employees with RTW after sickness absence. This type of work-directed intervention has been shown to be moderately effective in reducing sickness absence of people with depression in a Cochrane review [32].

Similar to previous research, employers felt larger organisations would be more able to make appropriate work accommodations and enable redeployment [22, 23]. On the other hand there was also a perception that small, family-run businesses may be more accommodating due to a stronger and more considerate relationship with employees.

On the theme of disclosure, viewpoints of employers and people with chronic pain were diverse in the current study. Previous research investigating the challenges faced by people with persistent pain in maintaining productive employment concluded that participants found weighing up the risks and benefits of disclosing their pain difficult, but less so if they were aware of support available [33]. Our study extends this by exploring disclosure in the context of job-seeking for people with chronic pain but reluctance to disclose for people with arthritis in this situation has been reported previously [34].

### Strengths and limitations

A strength of this study was that we included both people with chronic pain and employers, as the literature on employer views has hitherto been lacking. Engaging workplace representatives, particularly of medium-sized organisations, proved challenging, similar to previous research [35]. The most successful strategies were approaching people in organisations known professionally or personally to the research team through being involved in other research on work and health. The views of people in these organisations may be qualitatively different from those at other organisations. Further research is needed to collect a broader sample of employers’ views.

Notwithstanding our range of employers, one strength may be the range of people interviewed with different types of chronic pain who were unemployed, off sick or self-employed and the variety of employers from both public and private sectors and different size organisations.

We acknowledge the influence of the researchers’ role during data analysis phase and selection of themes for presentation. Reflexivity is an important tool used to analyse this influence [36]. Our backgrounds (SR- sociologist and MG, MU and RF - health professionals) will have influenced the research process. However, effective reflexive analysis was achieved by balancing self-awareness to increase insight with maintaining a primary focus on the interview data.

The credibility of this research is enhanced by investigator triangulation [37] where three researchers (MG, SR and RF) were involved in the data analysis and the choice of themes for presentation. Respondent validation [38] may have been useful for enhancing trustworthiness and while in our case resource and time prohibited the practice we acknowledge the limitation accordingly.

### Recommendations for the future

We recommend that the perceptions of people with chronic pain and employers need to be taken into consideration when designing and delivering RTW interventions. A collaborative intervention to tackle the obstacles and implement facilitators may be more beneficial for both improving quality of life in people unemployed with chronic pain, for employers in terms of finding an effective and productive workforce, as well as for the wider economy and health service more generally.

The feasibility of providing supported work placements to build confidence and enhance RTW opportunities for unemployed people with chronic pain could be tested. We hope that these results provide a useful starting point for the design of future interventions to help those with chronic pain to RTW.

Further research may also explore views in a larger more diverse set of employers and focus on exploring ways of creating opportunities for or starting discussions surrounding disclosure without fear of stigmatisation, or employer prejudice (either perceived or actual).

## Conclusions

People unemployed or off sick with chronic pain and employers have some common concerns about obstacles to RTW; these include lack of confidence and reliability. They are also in agreement about a number of ways to facilitate a successful RTW; these include making changes to the job and working conditions and providing access to interventions.

People unemployed with chronic pain and employers appear to differ in some of their other views, particularly about the disclosure of the pain condition and when this should occur. Designing interventions that incorporate factors that address the concerns of both potential employers and of those who are unemployed with chronic pain may help to improve the quality of interventions, and through doing so, improve both health and economic outcomes.

## Data Availability

The datasets generated and/or analysed during the current study are not publicly available due to (1) our having only permissions for anonymised summaries and quotations being presented in publications and (2) ethics approval not covering making these data publicly available, and (3) notwithstanding 1 and 2, risk of identification of participants from transcripts.

## Appendix

### Topic guides

Please note Topic Guides are dynamic documents that change with experience and data collected between interviews. This document is meant to be indicative only of the types of questions and topics that will be explored during the interview.

**Table 4:** Topic Guide - Qualitative Interviews - People with Chronic Pain

Topic	Questions prompts
About the participants chronic pain	I wonder if we might start by your telling me a little about your pain – the nature of it, how long you have had it and the ways it has affected your work life?
Perceived obstacles to return to work (physical/ psychosocial)	Have you worked previously? Could you tell me a little about what you did when you used to work? How did you come to stop working? I would be interested if you might tell me a little about the obstacles you perceive you have now to working? Have you previously found any adaptations, coping mechanisms, or sources of support that have helped you to work?
Disclosing chronic pain condition to employer	If a person discloses they have chronic pain on a job application form or during a job interview what impact do you think this might have on their chances of being employed? Did you ever have reservations about discussing your pain with your employer?
Perceived facilitators/ enablers of return to work	What do you think would enable people with chronic pain return to work?
Reasonable adjustments	Can you think of any workplace adaptations that in your case would help you be able to work/ maintain employment? Did any of your previous employers make workplace adjustments? Did these help?

**Table 5:** Topic Guide – Qualitative Interviews - Employers

Topic	Questions/prompts
Perceived obstacles to return to work for people with chronic pain (physical and psychosocial)	What do you think are the key things that stop people with chronic pain working/ or causes them to give up work or prevents them from returning to work after a period of absence?
Disclosure of chronic pain condition to employer	If people disclose they have chronic pain on a job application form or during an interview, are there any ways this might affect their chances of being employed?
Employing people with chronic pain conditions	What concerns might you have about employing someone who has a chronic pain condition?
Perceived facilitators of return to work for people with chronic pain	What do you think are the key things that could facilitate and help maintain a return to work for people with chronic pain?
Reasonable adjustments and willingness to make these	What kind of reasonable adjustments do you think might be appropriate for an employee who has a chronic pain condition? Thinking of your current organisation how likely are you to be able to make these adjustments for an employee in your organisation? Are there any adjustments that you can think of that you think might be appropriate but that in practice might be difficult to make?

## Abbreviations

CEO: Chief Executive Officer
GP: General Practitioner
NHS: National Health Service
RTW: Return-to-work

## References

[1] Jackson T, Thomas S, Stabile V, Shotwell M, Han X, McQueen K (2016). A systematic review and meta-analysis of the global burden of chronic pain without clear etiology in low- and middle-income countries: trends in heterogeneous data and a proposal for new assessment methods. Anesth Analg.

[2] Fayaz A, Croft P, Langford RM, Donaldson LJ, Jones GT (2016). Prevalence of chronic pain in the UK: a systematic review and meta-analysis of population studies. BMJ Open.

[3] Toye F, Seers K, Allcock N, Briggs M, Carr E, Barker K (2016). A synthesis of qualitative research exploring the barriers to staying in work with chronic musculoskeletal pain. Disabil Rehabil.

[4] Vos T (2017). Global, regional, and national incidence, prevalence, and years lived with disability for 328 diseases and injuries for 195 countries, 1990-2016: a systematic analysis for the global burden of disease study 2016. Lancet.

[5] Kronborg C, Handberg G, Axelsen F (2009). Health care costs, work productivity and activity impairment in non-malignant chronic pain patients. Eur J Health Econ.

[6] Maniadakis Nikolaos, Gray Alastair (2000). The economic burden of back pain in the UK. Pain.

[7] Department of Work and Pensions DoH: Improving lives. The future of Work, Health and Disability. In*.*: HMSO; 2017.

[8] Johannes CB, Le TK, Zhou X, Johnston JA, Dworkin RH (2010). The prevalence of chronic pain in United States adults: results of an internet-based survey. J Pain.

[9] Grant M, O'Beirne-Elliman J, Froud R, Underwood M, Seers K (2019). The work of return to work. Challenges of returning to work when you have chronic pain: a meta-ethnography. BMJ Open.

[10] Nordqvist C, Holmqvist C, Alexanderson K (2003). Views of laypersons on the role employers play in return to work when sick-listed. J Occup Rehabil.

[11] Job Shop [http://www.coventry.gov.uk/info/153/employment_support/2514/job_shop_services_for_people_looking_for_work].

[12] Froud R, Patterson S, Eldridge S, Seale C, Pincus T, Rajendran D, Fossum C, Underwood M (2014). A systematic review and meta-synthesis of the impact of low back pain on people's lives. BMC Musculoskelet Disord.

[13] Ritchie J, Spencer L, Bryman A, Burgess RG (1994). Qualitative data analysis for applied policy research. Analysing qualitative data.

[14] Gale NK, Heath G, Cameron E, Rashid S, Redwood S. Using the framework method for the analysis of qualitative data in multi-disciplinary health research. BMC Med Res Methodol. 2013;13:117–7. 10.1186/1471-2288-13-117 PMC3848812 24047204

[15] Patel S, Greasley K, Watson PJ (2007). Barriers to rehabilitation and return to work for unemployed chronic pain patients: a qualitative study. Eur J Pain.

[16] Dionne C, Bourbonnais R, Frémont P, Rossignol M, Stock S, Laperrière È (2013). Obstacles to and facilitators of return to work after work-disabling Back pain: the Workers' perspective. J Occup Rehabil.

[17] Magnussen L, Nilsen S, Råheim M (2007). Barriers against returning to work--as perceived by disability pensioners with back pain: a focus group based qualitative study. Disabil Rehabil.

[18] Sjöström R, Melin-Johansson C, Asplund R, Alricsson M (2011). Barriers to and possibilities of returning to work after a multidisciplinary rehabilitation programme. A qualitative interview study. Work.

[19] Soeker MS, Wegner L, Pretorius B (2008). I'm going Back to work: Back injured clients' perceptions and experiences of their worker roles. Work.

[20] Fassier J-B, Durand M-J, Caillard J-F, Roquelaure Y, Loisel P (2015). Results of a feasibility study: barriers and facilitators in implementing the Sherbrooke model in France. Scand J Work Environ Health.

[21] Grataloup M, Massardier-Pilonchéry A, Bergeret A, Fassier JB (2016). Job restrictions for healthcare workers with musculoskeletal disorders: consequences from the Superior’s viewpoint. J Occup Rehabil.

[22] Soklaridis S, Ammendolia C, Cassidy D (2010). Looking upstream to understand low back pain and return to work: psychosocial factors as the product of system issues. Soc Sci Med.

[23] Williams-Whitt K, Kristman V, Shaw WS, Soklaridis S, Reguly P (2016). A model of supervisor decision-making in the accommodation of workers with low Back pain. J Occup Rehabil.

[24] Wynne-Jones G, Buck R, Porteous C, Cooper L, Button LA, Main CJ, Phillips CJ (2011). What happens to work if you're unwell? Beliefs and attitudes of managers and employees with musculoskeletal pain in a public sector setting. J Occup Rehabil.

[25] Pransky GS, Fassier J-B, Besen E, Blanck P, Ekberg K, Feuerstein M, Munir F, Amick BC, Anema JR, Besen E (2016). Sustaining Work Participation Across the Life Course. J Occup Rehabil.

[26] Garthwaite K (2011). ‘The language of shirkers and scroungers?’ Talking about illness, disability and coalition welfare reform. Disability & Society.

[27] The Equality Act [http://www.legislation.gov.uk/ukpga/2010/15/contents].

[28] Coole C, Radford K, Grant M, Terry J (2013). Returning to work after stroke: perspectives of employer stakeholders, a qualitative study. J Occup Rehabil.

[29] Kalsi P, Turkistani W, Sykes C, Lucas A, Zarnegar R (2016). "work is a beautiful thing." exploring attitudes towards employment in chronic pain (CP) patients attending a pain management programme (PMP). J Vocational Rehab.

[30] Lundberg Tove, Melander Stina (2019). Key Push and Pull Factors Affecting Return to Work Identified by Patients With Long-Term Pain and General Practitioners in Sweden. Qualitative Health Research.

[31] Coole C, Watson PJ, Drummond A (2010). Low back pain patients' experiences of work modifications; a qualitative study. BMC Musculoskelet Disord.

[32] Nieuwenhuijsen K, Faber B, Verbeek JH, Neumeyer-Gromen A, Hees HL, Verhoeven AC, van der Feltz-Cornelis CM, Bültmann U. Interventions to improve return to work in depressed people. Cochrane Database Syst Rev. 2014;12. 10.1002/14651858.CD006237.pub3 25470301

[33] Oakman J, Kinsman N, Briggs AM (2017). Working with persistent pain: an exploration of strategies utilised to stay productive at work. J Occup Rehabil.

[34] Gignac MA, Cao X (2009). "should I tell my employer and coworkers I have arthritis?" a longitudinal examination of self-disclosure in the work place. Arthritis Rheum.

[35] Coole C, Nouri F, Narayanasamy M, Baker P, Khan S, Drummond A (2018). Engaging workplace representatives in research: what recruitment strategies work best?. Occup Med.

[36] Finlay L (2002). “Outing” the researcher: the provenance**,** Process, and Practice of Reflexivity. Qual Health Res.

[37] Denzin N: Sociological methods: a sourcebook, 5th edn. Chicago: Aldine Transaction; 1978.

[38] Birt L, Scott S, Cavers D, Campbell C, Walter F (2016). Member checking: a tool to enhance trustworthiness or merely a nod to validation?. Qual Health Res.

